# Chronic Q Fever Diagnosis—Consensus Guideline versus Expert Opinion

**DOI:** 10.3201/eid2107.130955

**Published:** 2015-07

**Authors:** Linda M. Kampschreur, Marjolijn C.A. Wegdam-Blans, Peter C. Wever, Nicole H.M. Renders, Corine E. Delsing, Tom Sprong, Marjo E.E. van Kasteren, Henk Bijlmer, Daan Notermans, Jan Jelrik Oosterheert, Frans S. Stals, Marrigje H. Nabuurs-Franssen, Chantal P. Bleeker-Rovers, on behalf of the Dutch Q Fever Consensus Group

**Affiliations:** Jeroen Bosch Hospital, ’s-Hertogenbosch, the Netherlands (L.M. Kampschreur, P.C. Wever, N.H.M. Renders);; University Medical Center Utrecht, Utrecht, the Netherlands (L.M. Kampschreur, J.J. Oosterheert);; Laboratory for Pathology and Medical Microbiology, Veldhoven, the Netherlands (M.C.A. Wegdam-Blans);; Radboud University Medical Center, Nijmegen, the Netherlands (C.E. Delsing, C.P. Bleeker-Rovers);; Canisius-Wilhelmina Ziekenhuis, Nijmegen (T. Sprong);; Canisius-Wilhelmina Ziekenhuis, Nijmegen (T. Sprong, M.H. Nabuurs-Franssen);; St. Elisabeth Hospital, Tilburg, the Netherlands (M.E.E. van Kasteren);; National Institute for Public Health and the Environment, Bilthoven, the Netherlands (H. Bijlmer, D. Notermans);; Atrium Medical Centre, Heerlen, the Netherlands (F.S. Stals)

**Keywords:** chronic Q fever, endocarditis, vascular infection, diagnosis, C. burnetii

## Abstract

Chronic Q fever, caused by C*oxiella burnetii*, has high mortality and morbidity rates if left untreated. Controversy about the diagnosis of this complex disease has emerged recently. We applied the guideline from the Dutch Q Fever Consensus Group and a set of diagnostic criteria proposed by Didier Raoult to all 284 chronic Q fever patients included in the Dutch National Chronic Q Fever Database during 2006–2012. Of the patients who had proven cases of chronic Q fever by the Dutch guideline, 46 (30.5%) would not have received a diagnosis by the alternative criteria designed by Raoult, and 14 (4.9%) would have been considered to have possible chronic Q fever. Six patients with proven chronic Q fever died of related causes. Until results from future studies are available, by which current guidelines can be modified, we believe that the Dutch literature-based consensus guideline is more sensitive and easier to use in clinical practice.

## Abstract 2

Literature-based consensus guideline is more sensitive and easier to use in clinical practice.

*Coxiella burnetii* is the causative agent of Q fever, a zoonosis occurring worldwide (*1*). Recently, a large epidemic occurred in the Netherlands with >4,000 cases of acute Q fever notified from 2007 through 2010 (*2*,*3*). Chronic Q fever develops in an estimated 1%–5% of all infected humans and can become manifest even years after primary infection (*1*,*4*). Endocarditis and infection in aneurysms or vascular prostheses are the most common manifestations (*1*,*5*,*6*). Untreated chronic Q fever has a poor prognosis, with a reported mortality rate of up to 60% (*1*,*7*). Adequate antibiotic treatment reduces the mortality rate for Q fever endocarditis to <5% (*7*). Treatment preferably consists of a combination of doxycycline and hydroxychloroquine for at least 18 months (nonprosthetic infection) to 24 months (prosthetic infection) and is recommended to be continued in case of unfavorable clinical or serologic response (*7*,*8*). Antibiotic guidelines for vascular chronic Q fever are not yet available, but antibiotic regimes for Q fever endocarditis have been applied to this disease entity as well. Early surgical intervention, with removal of infected material, might improve the prognosis of vascular chronic Q fever (*6*,*9*).

In the early course of chronic Q fever, most patients are asymptomatic or experience nonspecific symptoms such as low-grade fever, night sweats, and weight loss (*1*,*4*,*6*,*7*). In the case of endocarditis, findings on echocardiograph are often nonspecific or absent, which makes the diagnosis of chronic Q fever challenging (*7*). A PCR positive for *C. burnetii* or culture of the organism in blood or tissue, in the absence of acute Q fever, is a strong indicator for chronic Q fever. However, sensitivity on blood samples is only 50%–60% for both PCR and culture in patients with chronic Q fever (*10*,*11*). Therefore, serologic testing is also valuable for the diagnosis of chronic Q fever. A phase I IgG cutoff titer of 1:800, which is based on an in-house–developed immunofluorescence assay (IFA), has been internationally accepted for the diagnosis of chronic Q fever and is included in the modified Duke criteria for diagnosis of endocarditis (*12*,*13*). In the Netherlands, a commercial IFA (Focus Diagnostics, Inc., Cypress, CA, USA) is primarily used, with a proposed IgG cutoff value of 1:1,024 for chronic Q fever (*14*). Yet, recent studies show that serology results alone are not sufficient for the diagnosis of chronic Q fever, but that they should be combined with clinical data (*15*).

## Dutch Consensus Guideline

Faced with a large Q fever outbreak in the Netherlands and a rising number of (presumed) chronic Q fever patients, we were not able to find answers to all our questions about this complex disease in the literature. Moreover, randomized trials on diagnosis and treatment of this disease were lacking, and available data were not all applicable to the Dutch situation. For example, we found far more vascular localizations of chronic Q fever, with often severe complications, than had been described previously. Therefore, the Dutch Q Fever Consensus Group was initiated in 2010, in which diagnosis and subsequent treatment consequences for suspected chronic Q fever were discussed. We performed a thorough literature review and constructed a new guideline for the diagnosis of chronic Q fever, differentiating between proven, probable, and possible chronic Q fever (Table 1). We added advice for treatment and follow-up regimes for these 3 groups of patients. Antibiotic treatment and, if indicated, surgical treatment are recommended for all patients with proven chronic Q fever. The decision to start antibiotic treatment in patients with probable chronic Q fever depends on clinical characteristics and the condition of the patient, and should be determined by a multidisciplinary team. For possible chronic Q fever patients, antibiotic treatment should not be initiated, but follow-up is indicated.

**Table 1:** Dutch consensus guideline on chronic Q fever diagnostics*

Proven chronic Q fever	Probable chronic Q fever	Possible chronic Q fever
1. Positive *Coxiella burnetii* PCR of blood or tissue†	IFA ≥1:1,024 for *C. burnetii* phase I IgG‡	IFA ≥ 1:1,024 for *C. burnetii* phase I IgG‡ without manifestations meeting the criteria for proven or probable chronic Q fever
OR	AND any of the following:	
2. IFA ≥1:800 or 1:1,024 for *C. burnetii* phase I IgG†	Valvulopathy not meeting the major criteria of the modified Duke criteria (*13*)	
AND	Known aneurysm and/or vascular or cardiac valve prosthesis without signs of infection by means of TEE/ TTE, FDG-PET, CT, MRI, or AUS	
Definite endocarditis according to the modified Duke criteria (*13*)		
OR		
Proven large vessel or prosthetic infection by imaging studies (FDG-PET, CT, MRI, or AUS)	Suspected osteomyelitis or hepatitis as manifestation of chronic Q fever	
	Pregnancy	
	Symptoms and signs of chronic infection, such as fever, weight loss and night sweats, hepato-splenomegaly, persistent raised ESR and CRP	
	Granulomatous tissue inflammation, proven by histological examination	
	Immunocompromised state	

*Source (*14*). IFA, immunofluorescence assay; TEE, transesophageal echocardiography; TTE, transthoracic echocardiography; FDG-PET, fluorodeoxyglucose positron emission tomography; CT, computed tomography; MRI, magnetic resonance imaging; AUS, abdominal ultrasound; ESR, erythrocyte sedimentation rate; CRP, C-reactive protein. †In the absence of acute infection. ‡Cut-off depends on the IFA technique used, whether in-house developed or commercial.

After the Dutch consensus guideline was reported (*14*), a reaction by French researcher Didier Raoult was published; he did not agree with this proposed guideline and formulated alternative diagnostic criteria on the basis of his expert opinion (Table 2) (*16*). Professor Raoult is the undisputed leading authority on Q fever, and his opinion and the scientific publications from his research group should be considered by anyone working in the field of Q fever. Here, we attempt to resolve these differences of opinion by applying both criteria to cases from the Dutch National Chronic Q Fever Database.

**Table 2:** Diagnostic guideline for chronic Q fever proposed by Raoult*

Q fever endocarditis
A. Definite criteria
Positive culture, PCR, or immunochemistry of a cardiac valve
B. Major criteria
Microbiology: positive culture or PCR of the blood or an emboli or serology with IgG I antibodies ≥6,400
Evidence of endocardial involvement:
Echocardiogram positive for IE: oscillating intra-cardiac mass on valve or supporting structure, in the path of regurgitant jets, or on implanted material in the absence of an alternative anatomic explanation; or abscess; or new partial dehiscence of prosthetic vale; or new valvular regurgitation (worsening or changing of pre-existent murmur not sufficient)
PET scan showing a specific valve fixation and mycotic aneurysm
C. Minor criteria
Predisposing heart condition (known or found on echocardiograph)
Fever, temperature >38°C
Vascular phenomena, major arterial emboli, septic pulmonary infarcts, mycotic aneurysm (see at PET scan), intracranial hemorrhage, conjunctival hemorrhages, and Janeway lesions
Immunologic phenomena: glomerulonephritis, Osle nodes, Roth spots, or rheumatoid factor
Serologic evidence: IgG I antibodies ≥800 <6,400
Diagnosis definite
1. 1A criterion
2. 2B criterion
3. 1B, and 3C criterion
Diagnosis possible
1. 1B criterion, 2C criteria (including microbiology evidence, and cardiac predisposition)
2. 3C criteria (including positive serology, and cardiac predisposition)
Q fever vascular infection
A. Definite criteria
Positive culture, PCR, or immunochemistry of an arterial sample (prosthesis or aneurysm) or a periarterial abscess or a spondylodiscitis linked to aorta
B. Major criteria
Microbiology: positive culture or PCR of the blood or an emboli or serology with IgG I antibodies ≥6,400
Evidence of vascular involvement CT scan: aneurysm or vascular prosthesis + periarterial abscess, fistula, or spondylodiscitis PET scan: specific fixation on an aneurysm or vascular prosthesis
C. Minor criteria
Serological IgG I ≥800 <6,400
Fever, temperature >38°C
Emboli
Underlying vascular predisposition (aneurysm or vascular prosthesis)
Diagnosis definite
1. 1A criterion
2. 2B criterion
3. 1B and 2C criterion (including microbiology findings and vascular predisposition)
Diagnosis possible
Vascular predisposition, serological evidence and fever or emboli
*Source (*16*). IE, infective endocarditis; PET, positron emission tomography; IFA, immunofluorescence assay; CT, computed tomography.

## Dutch Consensus Guideline versus Expert Opinion Guideline

A critical difference in the diagnostic criteria proposed by Raoult and those of the Dutch Q Fever Consensus Group is the diagnostic value attributed to *C. burnetii* PCR positivity of blood samples. Because we are unaware of clinical entities, other than acute and chronic Q fever, for which a PCR positive for *C. burnetii* in blood would be exhibited, we believe that positive blood PCR findings, in the absence of acute Q fever, prove chronic Q fever. The alternative criteria, on the other hand, state that a positive PCR finding in blood should be accompanied by a clear endocarditis focus shown on echocardiograph, a clear vascular focus on imaging studies, or at least 2 or 3 “minor criteria” (Table 2). Moreover, the alternative criteria attribute great value to the phase I IgG titer, proposing a phase I IgG ≥1:6,400 as a major criterion for Q fever endocarditis and Q fever vascular infection, in contrast to a phase I IgG ≥1:800 and <1:6,400 proposed as a minor criterion. This proposal contradicts the internationally accepted modified Duke criteria, which state that a phase I IgG ≥1:800 is a major criterion for infective (Q fever) endocarditis (*13*).

The alternative criteria also generally oppose the term chronic Q fever but makes a distinction in 2 manifestations: Q fever endocarditis and Q fever vascular infection. More rare manifestations, such as pericarditis, hepatitis, and osteomyelitis, are left undefined, however.

After the recent outbreak of Q fever in the Netherlands, we initiated the Dutch National Chronic Q Fever Database, a joint effort by multiple hospitals in areas affected by Q fever, to monitor all chronic Q fever cases in the Netherlands. All hospitals with chronic Q fever patients, also outside the notified Q fever–epidemic areas, were actively approached. Design of the database and use of the collected information for analysis and scientific publications were approved by the Medical Research Ethics Committee of the University Medical Center Utrecht in Utrecht, the Netherlands. Part of these data had previously been published in a report discussing serologic profiles of patients with chronic Q fever (*15*).

Until the end of May 2012, a total of 284 patients had been included in our database (although the epidemic started in 2009, all patients from 2006 on are included): 151 patients (53.2%) had proven chronic Q fever, 64 patients (22.5%) had probable chronic Q fever, and 69 patients (24.3%) had possible chronic Q fever, according to the Dutch consensus guideline. We reevaluated these chronic Q fever cases with the alternative diagnostic criteria (Table 3). Of the case-patients with proven chronic Q fever according to the Dutch guideline, 46 (30.5%) would have been left undiagnosed with the alternative criteria. For cases of probable chronic Q fever, 58 cases (90.6%) would have been undiagnosed, and for possible cases of chronic Q fever, all 69 cases.

**Table 3:** Comparison of chronic Q fever diagnosis according to the Dutch consensus guideline* and the alternative criteria†

Alternative criteria	Dutch consensus chronic Q fever guideline		
	Proven, no. (%), n =151	Probable, no. (%), n = 64	Possible, no (%), n = 69
Definite Q fever endocarditis	21 (13.9)	0	0
Possible Q fever endocarditis	8 (5.3)	4 (6.3)	0
Definite Q fever vascular infection	76 (50.3)	0	0
Possible Q fever vascular infection	0	2 (3.1)	0
No diagnosis of chronic Q fever	46 (30.5)	58 (90.6)	69 (100.0)

*Source (*14*). †Source (*16*).

The conditions of 8 patients with proven chronic Q fever (based on PCR positivity for *C. burnetii* in blood and suspicion of endocarditis) would have been diagnosed with possible Q fever endocarditis only by the alternative criteria (Table 4). Eighteen patients with proven chronic Q fever would not have been diagnosed with Q fever endocarditis at all, because echocardiography results did not match any major clinical Duke criterion, as is often observed in cases of Q fever endocarditis (*7*). Of the 8 patients with proven chronic Q fever endocarditis (by Dutch consensus guideline) who had been given a diagnosis of possible endocarditis according to the alternative criteria, 2 patients would have been considered to have definite endocarditis by the modified Duke criteria (*13*).

**Table 4:** Characteristics and outcome of patients diagnosed with chronic Q fever using the Dutch consensus guideline* but without (definite) chronic Q fever according to alternative criteria†

Dutch consensus guideline	Alternative criteria	
	Possible Q fever endocarditis or vascular infection, no. (%), n =14	No diagnosis, no. (%), n = 173
Proven Q fever	8 (57.1)	46 (26.6)
Endocarditis	8 (57.1)	18 (10.4)^;^
PCR positive for *Coxiella burnetii* in blood	6 (42.9)	18 (10.4)
Evidence of endocardial involvement	2 (14.3)	0
Vascular infection	0	24 (13.9)‡
PCR positive in blood	0	7 (4.0)
Vascular focus on imaging	0	17 (9.8)
Other or no focus§	0	7 (4.1)
Deceased	2 (14.3)	8 (4.6)
Death probably due to Q fever	2 (14.3)	4 (2.3)¶
Probable Q fever	6 (42.9)	58 (33.5)
Endocarditis	4 (28.6)	22 (12.7)
Vascular infection	2 (14.3)	16 (9.3)
Other or no focus	0	20 (11.6)
Deceased	2 (14.3)	4 (2.3)
Death probably due to Q fever	1 (7.1)	0
Possible Q fever	0	69 (39.9)

*Source (*14*). †Source (*16*). ‡In 3 patients with proven chronic Q fever, imaging studies showed that the focus of infection was in both the heart valves and the vascular structures. §All were PCR positive. ¶For 2 patients, PCR of vascular and heart valve tissue obtained at autopsy was positive for *C. burnetii*.

Twenty-four patients with a vascular *C. burnetii* infection (Dutch consensus guideline) would not have been diagnosed with chronic Q fever by using the alternative criteria (Table 4). Seventeen of these patients had a positive vascular lesion on fluorodeoxyglucose–positron emission tomography/computed tomography (FDG-PET/CT) with phase I IgG ≥1:800 and <1:6,400. Seven patients had a PCR of blood positive for *C. burnetii*, in combination with an aneurysm or vascular prosthesis but no signs of infection on FDG-PET/CT. According to the Dutch consensus guideline, there were 5 patients with proven chronic Q fever with no known focus and 2 patients with Q fever with a focus other than endocarditis or vascular infection who would have been missed by using the alternative criteria. Five (repeatedly) had a positive *C. burnetii* PCR of blood but no clear infectious focus on echocardiograph and FDG-PET/CT scan. One patient had a positive PCR in blood with clinical pericarditis, and 1 patient had a positive PCR in blood during pregnancy with phase I IgG >1:1,024 and a positive PCR of placental tissue.

Notably, 10 patients with cases of proven chronic Q fever that were not diagnosed as definite chronic Q fever by the alternative criteria died (2 with possible chronic Q fever and 8 without chronic Q fever according to the alternative guideline). Six of these patients died due to clear chronic Q fever–related manifestations (2 with possible chronic Q fever and 4 without chronic Q fever according to the alternative guideline, Table 4). The 2 patients with possible chronic Q fever died of complications caused by endocarditis, one had a double-pathogen endocarditis with *Staphylococcus aureus*. Two of the 4 patients without chronic Q fever according to the alternative guideline died due to aortoduodenal fistula, both with a phase I IgG >1:1024, but <1:6400, negative PCR on blood, and a clear FDG-positive vascular focus on PET/CT. In 1 of these 2 patients, Q fever vascular infection was confirmed postmortem with a positive PCR of the abdominal aortic aneurysm. No autopsy was performed on the other patient, unfortunately. The third patient, who had a history of a biologic heart valve replacement, an FDG-PET/CT negative aortic aneurysm, and a positive *C. burnetii* PCR of blood, eventually died of heart failure. Postmortem analysis demonstrated that PCR of the heart valve confirmed *C. burnetii* infection and thus Q fever endocarditis. Another chronic Q fever patient with positive PCR results of blood and minor valve lesions, according to the Duke criteria, died of gastrointestinal bleeding, probably due to aorto-intestinal fistula.

## Conclusions

Several major differences exists between the Dutch consensus guideline for the diagnosis of chronic Q fever and the alternative criteria. These alternative criteria define only Q fever endocarditis and Q fever vascular infection and oppose the term chronic Q fever. However, this distinction is not accompanied by therapeutic consequences for each of these manifestations, which we believe makes these guidelines less practical.

It must be acknowledged that, because all patients included in our study met the Dutch criteria for proven, probable, or possible chronic Q fever, other guidelines can only perform with less accuracy in comparison. Nevertheless, sensitivity of the Dutch guideline is markedly higher than with the alternative criteria: ≈31% of proven chronic Q fever case-patients would have been missed as well as almost all patients with probable and possible cases, including at least 4 patients who eventually died of chronic Q fever–related causes. Specificity of the Dutch consensus guideline is probably lower than that of the alternative criteria, but because mortality and morbidity rates are high when chronic Q fever cases are untreated, we believe sensitivity is of greater importance in clinical practice. Our data illustrate that, when proven cases of chronic Q fever are missed, and patients are therefore not adequately treated, these patients are at high risk for severe complications and death.

As stated before, the most critical difference between the criteria of the Dutch guideline and those of the alternative guideline is the acknowledgment of a positive *C. burnetii* PCR as a marker of proven chronic Q fever in the absence of acute Q fever. Of course, this difference should be interpreted with care. In our opinion, patients without endocarditis or vascular infection on imaging studies but with a positive PCR in blood should also be treated for chronic Q fever, as they may suffer from not yet clinically visible endocarditis or vascular infection, which was confirmed by the postmortem results of 2 of our patients described above. A single positive *C. burnetii* PCR of blood is highly suggestive for chronic Q fever when acute Q fever is excluded. A PCR test will not be performed in patients without symptoms and without any risk factors for chronic Q fever, so cases in whom a positive PCR is the only factor indicating chronic Q fever is a theoretical consideration. We have observed few patients, in the absence of signs of acute Q fever, with elevated phase I IgG titers not fulfilling the serologic criteria of chronic Q fever (phase I IgG ≥1:800 or 1,024) but with a positive *C. burnetii* PCR of blood or tissue. In these cases, we are convinced that PCR-positivity proves chronic Q fever. No patients in our chronic Q fever database who had a positive PCR on blood or tissue had a phase I IgG titer of ≤1:256.

We agree with the statement that proven chronic Q fever will not develop in some patients with probable chronic Q fever and in most patients with possible chronic Q fever. We therefore do not advocate treating all of these patients with long-term antibacterial drugs. Nevertheless, we do think that these patients should all be examined for a chronic Q fever focus and should continue to be monitored closely, at least until further research offers more clarity regarding the prognosis of these patients. If these patients do not receive a diagnosis of possible or probable chronic Q fever, they might not receive such close follow-up. Moreover, the Dutch consensus guideline is easier to use, adds treatment advice, and also applies to patients with chronic Q fever manifestations that are rarer than endocarditis and vascular infection.

We hope that, with the future results from the Dutch National Chronic Q Fever Database and joint efforts of international researchers and experts in the field of Q fever, these guidelines can be modified to provide definite evidence-based criteria for diagnosis and treatment of this complex disease. In the meantime, the Dutch consensus guideline created on the basis of the scarce available literature is, in our opinion, safer and easier to use in clinical practice than the alternative expert-based criteria.
